# Supplementary material for: Trends in Antimicrobial Resistance of Uropathogens Isolated from Urinary Tract Infections in a Tertiary Care Hospital in Dhaka, Bangladesh
Source: Antibiotics (Basel). 2024 Sep 27;13(10):925. doi: 10.3390/antibiotics13100925 (PMC11505449; doi:10.3390/antibiotics13100925)
Supplement: Supplementary file 1 [file antibiotics-13-00925-s001.zip › antibiotics-3167039-supplementary.pdf]

## Supplementary Material

**Table S1.** Frequency of uropathogens isolated from patients with UTI

| Name of the Isolate | No of the Isolates |
|---|---|
| *E. coli* | 544 |
| *Klebsiella* spp. | 143 |
| *Enterococcus* spp. | 78 |
| *Candida* spp. | 58 |
| *Enterobacter* spp. | 34 |
| *Pseudomonas* spp. | 9 |
| *Proteus* spp. | 8 |
| *Staphylococcus aureus* | 7 |
| *Acinetobacter* spp. | 5 |
| *Acetobacter* spp. | 2 |
| *Citrobacter* spp. | 1 |
| *Lactobacillus* spp. | 1 |
| *Morganella morganii* | 1 |
| Polymicrobial Growth | 110 |
| **Total** | **1001** |

**Table S2.** Antibiotic resistance profile of bacteria isolated from patients positive for UTI. The table shows the number and percentage of antibiotic-resistant bacterial isolates for each antibiotic. Bacterial isolates include- Gram-negative*: E. coli, Klebsiella* spp., *Enterobacter* spp., *Pseudomonas* spp., *Proteus* spp., *Acinetobacter* spp., *Acetobacter* spp., *Citrobacter* spp., *Morganella morganii* and Gram-positive*: Enterococcus* spp., *Staphylococcus aureus*, and *Lactobacillus* spp.

| Antibiotic | Number of resistant isolates (%) | | | | | | | | | | | |
|---|---|---|---|---|---|---|---|---|---|---|---|---|
| | Gram –ve Bacteria | | | | | | | | | Gram +ve Bacteria | | |
| | *E. coli* (*n*=544) | *Klebsiella* spp. (*n*=143) | *Enterobacter* spp. (*n*=34) | *Pseudomonas* spp. (*n*=9) | *Proteus* spp. (*n*=8) | *Acinetobacter* spp. (*n*=5) | *Acetobacter* spp.(*n*=2) | *Citrobacter* spp. (*n*=1) | *Morganella morganii* (*n*=1) | *Enterococcus* spp. (*n*=78) | *Staphylococcus aureus* (*n*=7) | *Lactobacillus* spp.(*n*=1) |
| **Cefuroxime** | 352 (64.71%) | 67 (46.85%) | 17 (50.00%) | 9 (100%) | 3 (37.50%) | 2 (40.00%) | 1 (50.00%) | 0 (0%) | 1 (100%) | 47 (60.26%) | 0 (0%) | 1 (100%) |
| **Ceftazidime** | 351 (64.71%) | 67 (46.85%) | 19 (55.88%) | 9 (100%) | 3 (37.50%) | 2 (40.00%) | 1 (50.00%) | 1 (100%) | 0 (0%) | 58 (74.36%) | ND | 1 (100%) |
| **Amoxicillin-clavulanate** | 338 (62.13%) | 64 (44.76%) | 16 (47.06%) | 2 (22.22%) | 1 (12.50%) | 2 (40.00%) | 1 (50.00%) | 1 (100%) | 0 (0%) | 54 (69.23%) | ND | 1 (100%) |
| **Netilmicin** | 329 (60.48%) | 70 (48.95%) | 25 (73.53%) | 7 (77.78%) | 3 (37.50%) | 2 (40.00%) | 1 (50.00%) | 1 (100%) | 1 (100%) | 37 (47.44%) | 0 (0%) | 1 (100%) |
| **Trimethoprim-Sulfamethoxazole** | 316 (58.09%) | 69 (48.25%) | 15 (44.12%) | 2 (22.22%) | 3 (37.50%) | 1 (20%) | 1 (50.00%) | 1 (100%) | 0 (0%) | 70 (89.74%) | 0 (0%) | 1 (100%) |
| **Levofloxacin** | 312 (57.35%) | 58 (40.56%) | 13 (38.24%) | 7 (77.78%) | 1 (12.50%) | 1 (20%) | 1 (50.00%) | 0 (0%) | 0 (0%) | 50 (64.10%) | ND | 1 (100%) |
| **Ciprofloxacin** | 306 (56.25%) | 63 (44.06%) | 16 (47.06%) | 7 (77.78%) | 1 (12.50%) | 1 (20%) | 1 (50.00%) | 0 (0%) | 0 (0%) | 44 (56.41%) | ND | 1 (100%) |
| **Aztreonam** | 303 (55.70%) | 47 (32.87%) | 10 (29.41%) | 4 (44.44%) | 2 (25.00%) | 2 (40.00%) | 1 (50.00%) | 1 (100%) | 1 (100%) | 41 (52.56%) | 2 (28.57%) | 0 (0%) |
| **Cefpodoxime** | 275 (50.55%) | 55 (38.46%) | 9 (26.47%) | 8 (88.89%) | 4 (50.00%) | 2 (40.00%) | 2 (100%) | 1 (100%) | 1 (100%) | 27 (34.62%) | 2 (28.57%) | 0 (0%) |
| **Cefixime** | 126 (23.16%) | 42 (29.37%) | 17 (50.00%) | 8 (88.89%) | 7 (87.50%) | 2 (40.00%) | 1 (50.00%) | 1 (100%) | 0 (0%) | 11 (14.10%) | 0 (0%) | 1 (100%) |
| **Amikacin** | 21 (3.86%) | 10 (6.99) | 5 (14.71%) | 4 (44.44%) | 0 (0%) | 1 (20%) | 1 (50.00%) | 1 (100%) | 0 (0%) | 59 (75.64%) | ND | 0 (0%) |

[1] ND=Resistance profiles of *Staphylococcus aureus* for ceftazidime, amoxicillin-clavulanate, levofloxacin, ciprofloxacin and amikacin were not determined.

**Table S3.** Trends in resistance rates of *E. coli*, *Klebsiella* spp., *Enterobacte*r spp. and *Enterococcus* spp. over time (covering all months of 2018 except August).

| Bacteria | Months | Total no. of resistant bacteria isolated each month | Resistant bacteria to Antibiotic | | | | | | | | | | | | | | | | | | | | | |
|---|---|---|---|---|---|---|---|---|---|---|---|---|---|---|---|---|---|---|---|---|---|---|---|---|
| | | | Amikacin | | Netilmicin | | Trimethoprim-sulfamethoxazole | | Amoxicillin-clavulanate | | Ceftazidime | | Aztreonam | | Ciprofloxacin | | Levofloxacin | | Cefuroxime | | Cefixime | | Cefpodoxime | |
| | | | Frequency (n) | Percentage (%) | Frequency (n) | Percentage (%) | Frequency (n) | Percentage (%) | Frequency (n) | Percentage (%) | Frequency (n) | Percentage (%) | Frequency (n) | Percentage (%) | Frequency (n) | Percentage (%) | Frequency (n) | Percentage (%) | Frequency (n) | Percentage (%) | Frequency (n) | Percentage (%) | Frequency (n) | Percentage (%) |
| *E. coli* | January | 36 | 1 | 2.78 | 2 | 5.56 | 20 | 55.56 | 29 | 80.56 | 22 | 61.11 | 23 | 63.89 | 13 | 36.11 | 12 | 33.33 | 26 | 72.22 | 25 | 69.44 | 24 | 66.67 |
| | February | 61 | 2 | 3.28 | 49 | 80.33 | 40 | 65.57 | 38 | 62.30 | 39 | 63.93 | 37 | 60.66 | 40 | 65.57 | 40 | 65.57 | 41 | 67.21 | 10 | 16.39 | 31 | 50.82 |
| | March | 59 | 1 | 1.69 | 4 | 6.78 | 29 | 49.15 | 46 | 77.97 | 42 | 71.19 | 41 | 69.49 | 37 | 62.71 | 36 | 61.02 | 43 | 72.88 | 43 | 72.88 | 41 | 69.49 |
| | April | 64 | 5 | 7.81 | 51 | 79.69 | 40 | 62.50 | 40 | 62.50 | 46 | 71.88 | 40 | 62.50 | 44 | 68.75 | 41 | 64.06 | 45 | 70.31 | 8 | 12.50 | 31 | 48.44 |
| | May | 58 | 4 | 6.90 | 34 | 58.62 | 29 | 50.00 | 26 | 44.83 | 31 | 53.45 | 19 | 32.76 | 28 | 48.28 | 27 | 46.55 | 29 | 50.00 | 11 | 18.97 | 22 | 37.93 |
| | June | 46 | 2 | 4.35 | 31 | 67.39 | 23 | 50.00 | 26 | 56.52 | 25 | 54.35 | 23 | 50.00 | 24 | 52.17 | 21 | 45.65 | 26 | 56.52 | 5 | 10.87 | 27 | 58.70 |
| | July | 42 | 0 | 0.00 | 31 | 73.81 | 24 | 57.14 | 23 | 54.76 | 27 | 64.29 | 24 | 57.14 | 26 | 61.90 | 24 | 57.14 | 26 | 61.90 | 7 | 16.67 | 19 | 45.24 |
| | September | 68 | 0 | 0.00 | 49 | 72.06 | 42 | 61.76 | 40 | 58.82 | 45 | 66.18 | 42 | 61.76 | 43 | 63.24 | 40 | 58.82 | 43 | 63.24 | 11 | 16.18 | 37 | 54.41 |
| | October | 61 | 3 | 4.92 | 43 | 70.49 | 38 | 62.30 | 40 | 65.57 | 40 | 65.57 | 29 | 47.54 | 38 | 62.30 | 39 | 63.93 | 41 | 67.21 | 6 | 9.84 | 23 | 37.70 |
| | November | 44 | 3 | 6.82 | 32 | 72.73 | 28 | 63.64 | 27 | 61.36 | 31 | 70.45 | 22 | 50.00 | 13 | 29.55 | 28 | 63.64 | 29 | 65.91 | 0 | 0.00 | 18 | 40.91 |
| | December | 5 | 0 | 0.00 | 3 | 60.00 | 3 | 60.00 | 3 | 60.00 | 3 | 60.00 | 3 | 60.00 | 0 | 0.00 | 3 | 60.00 | 3 | 60.00 | 0 | 0.00 | 2 | 40.00 |
| *Klebsiella* spp. | January | 12 | 0 | 0.00 | 0 | 0.00 | 9 | 75.00 | 8 | 66.67 | 5 | 41.67 | 5 | 41.67 | 3 | 25.00 | 3 | 25.00 | 6 | 50.00 | 5 | 41.67 | 4 | 33.33 |
| | February | 10 | 1 | 10.00 | 6 | 60.00 | 5 | 50.00 | 5 | 50.00 | 5 | 50.00 | 3 | 30.00 | 5 | 50.00 | 5 | 50.00 | 5 | 50.00 | 5 | 50.00 | 6 | 60.00 |
| | March | 3 | 0 | 0.00 | 0 | 0.00 | 3 | 100.00 | 3 | 100.00 | 3 | 100.00 | 3 | 100.00 | 3 | 100.00 | 0 | 0.00 | 3 | 100.00 | 3 | 100.00 | 3 | 100.00 |
| | April | 8 | 0 | 0.00 | 4 | 50.00 | 2 | 25.00 | 1 | 12.50 | 2 | 25.00 | 2 | 25.00 | 2 | 25.00 | 2 | 25.00 | 2 | 25.00 | 1 | 12.50 | 1 | 12.50 |
| | May | 19 | 3 | 15.79 | 11 | 57.89 | 10 | 52.63 | 10 | 52.63 | 10 | 52.63 | 9 | 47.37 | 10 | 52.63 | 10 | 52.63 | 11 | 57.89 | 7 | 36.84 | 9 | 47.37 |
| | June | 15 | 2 | 13.33 | 8 | 53.33 | 5 | 33.33 | 5 | 33.33 | 7 | 46.67 | 4 | 26.67 | 5 | 33.33 | 5 | 33.33 | 5 | 33.33 | 2 | 13.33 | 4 | 26.67 |
| | July | 16 | 2 | 12.50 | 9 | 56.25 | 7 | 43.75 | 6 | 37.50 | 7 | 43.75 | 4 | 25.00 | 7 | 43.75 | 7 | 43.75 | 7 | 43.75 | 6 | 37.50 | 4 | 25.00 |
| | September | 9 | 1 | 11.11 | 7 | 77.78 | 6 | 66.67 | 5 | 55.56 | 6 | 66.67 | 5 | 55.56 | 6 | 66.67 | 5 | 55.56 | 6 | 66.67 | 5 | 55.56 | 9 | 100.00 |
| | October | 21 | 0 | 0.00 | 12 | 57.14 | 11 | 52.38 | 11 | 52.38 | 12 | 57.14 | 7 | 33.33 | 12 | 57.14 | 11 | 52.38 | 12 | 57.14 | 5 | 23.81 | 9 | 42.86 |
| | November | 6 | 0 | 0.00 | 4 | 66.67 | 5 | 83.33 | 5 | 83.33 | 5 | 83.33 | 4 | 66.67 | 4 | 66.67 | 5 | 83.33 | 5 | 83.33 | 1 | 16.67 | 6 | 100.00 |
| | December | 2 | 0 | 0.00 | 1 | 50.00 | 0 | 0.00 | 0 | 0.00 | 0 | 0.00 | 0 | 0.00 | 0 | 0.00 | 0 | 0.00 | 0 | 0.00 | 0 | 0.00 | 0 | 0.00 |
| *Enterobacter* spp. | January | 1 | 0 | 0.00 | 0 | 0.00 | 1 | 100.00 | 1 | 100.00 | 1 | 100.00 | 1 | 100.00 | 1 | 100.00 | 0 | 0.00 | 1 | 100.00 | 1 | 100.00 | 1 | 100.00 |
| | February | 7 | 2 | 28.57 | 4 | 57.14 | 4 | 57.14 | 3 | 42.86 | 4 | 57.14 | 1 | 14.29 | 4 | 57.14 | 3 | 42.86 | 4 | 57.14 | 2 | 28.57 | 1 | 14.29 |
| | March | 1 | 1 | 100.00 | 1 | 100.00 | 0 | 0.00 | 1 | 100.00 | 1 | 100.00 | 1 | 100.00 | 1 | 100.00 | 1 | 100.00 | 1 | 100.00 | 1 | 100.00 | 1 | 100.00 |
| | April | 5 | 0 | 0.00 | 4 | 80.00 | 3 | 60.00 | 2 | 40.00 | 4 | 80.00 | 1 | 20.00 | 2 | 40.00 | 2 | 40.00 | 2 | 40.00 | 3 | 60.00 | 1 | 20.00 |
| | May | 3 | 2 | 66.67 | 2 | 66.67 | 1 | 33.33 | 1 | 33.33 | 2 | 66.67 | 1 | 33.33 | 2 | 66.67 | 1 | 33.33 | 1 | 33.33 | 3 | 100.00 | 2 | 66.67 |
| | June | 6 | 5 | 83.33 | 4 | 66.67 | 6 | 100.00 | 5 | 83.33 | 5 | 83.33 | 1 | 16.67 | 4 | 66.67 | 4 | 66.67 | 4 | 66.67 | 0 | 0.00 | 1 | 16.67 |
| | July | 0 | 0 | 0.00 | 0 | 0.00 | 0 | 0.00 | 0 | 0.00 | 0 | 0.00 | 0 | 0.00 | 0 | 0.00 | 0 | 0.00 | 0 | 0.00 | 0 | 0.00 | 0 | 0.00 |
| | September | 1 | 0 | 0.00 | 1 | 100.00 | 0 | 0.00 | 0 | 0.00 | 1 | 100.00 | 1 | 100.00 | 0 | 0.00 | 0 | 0.00 | 1 | 100.00 | 1 | 100.00 | 1 | 100.00 |
| | October | 4 | 0 | 0.00 | 4 | 100.00 | 3 | 75.00 | 2 | 50.00 | 3 | 75.00 | 1 | 25.00 | 2 | 50.00 | 2 | 50.00 | 2 | 50.00 | 3 | 75.00 | 0 | 0.00 |
| | November | 1 | 0 | 0.00 | 1 | 100.00 | 1 | 100.00 | 1 | 100.00 | 1 | 100.00 | 1 | 100.00 | 1 | 100.00 | 1 | 100.00 | 1 | 100.00 | 0 | 0.00 | 1 | 100.00 |
| | December | 0 | 0 | 0.00 | 0 | 0.00 | 0 | 0.00 | 0 | 0.00 | 0 | 0.00 | 0 | 0.00 | 0 | 0.00 | 0 | 0.00 | 0 | 0.00 | 0 | 0.00 | 0 | 0.00 |
| *Enterococcus* spp. | January | 3 | 1 | 33.33 | 0 | 0.00 | 1 | 33.33 | 1 | 33.33 | 2 | 66.67 | 2 | 66.67 | 1 | 33.33 | 1 | 33.33 | 2 | 66.67 | 2 | 66.67 | 2 | 66.67 |
| | February | 6 | 5 | 83.33 | 2 | 33.33 | 6 | 100.00 | 4 | 66.67 | 4 | 66.67 | 3 | 50.00 | 2 | 33.33 | 3 | 50.00 | 2 | 33.33 | 1 | 16.67 | 3 | 50.00 |
| | March | 3 | 3 | 100.00 | 1 | 33.33 | 1 | 33.33 | 3 | 100.00 | 3 | 100.00 | 3 | 100.00 | 1 | 33.33 | 1 | 33.33 | 3 | 100.00 | 3 | 100.00 | 3 | 100.00 |
| | April | 14 | 10 | 71.43 | 9 | 64.29 | 12 | 85.71 | 9 | 64.29 | 9 | 64.29 | 9 | 64.29 | 9 | 64.29 | 9 | 64.29 | 8 | 57.14 | 1 | 7.14 | 5 | 35.71 |
| | May | 10 | 9 | 90.00 | 5 | 50.00 | 10 | 100.00 | 8 | 80.00 | 9 | 90.00 | 3 | 30.00 | 7 | 70.00 | 7 | 70.00 | 6 | 60.00 | 1 | 10.00 | 3 | 30.00 |
| | June | 3 | 0 | 0.00 | 2 | 66.67 | 1 | 33.33 | 2 | 66.67 | 1 | 33.33 | 1 | 33.33 | 2 | 66.67 | 2 | 66.67 | 2 | 66.67 | 1 | 33.33 | 1 | 33.33 |
| | July | 8 | 6 | 75.00 | 5 | 62.50 | 7 | 87.50 | 5 | 62.50 | 5 | 62.50 | 4 | 50.00 | 6 | 75.00 | 6 | 75.00 | 6 | 75.00 | 1 | 12.50 | 3 | 37.50 |
| | September | 1 | 1 | 100.00 | 0 | 0.00 | 1 | 100.00 | 1 | 100.00 | 1 | 100.00 | 1 | 100.00 | 0 | 0.00 | 1 | 100.00 | 1 | 100.00 | 0 | 0.00 | 1 | 100.00 |
| | October | 13 | 8 | 61.54 | 6 | 46.15 | 13 | 100.00 | 7 | 53.85 | 8 | 61.54 | 9 | 69.23 | 7 | 53.85 | 7 | 53.85 | 6 | 46.15 | 0 | 0.00 | 5 | 38.46 |
| | November | 1 | 0 | 0.00 | 1 | 100.00 | 1 | 100.00 | 1 | 100.00 | 1 | 100.00 | 1 | 100.00 | 1 | 100.00 | 1 | 100.00 | 1 | 100.00 | 0 | 0.00 | 1 | 100.00 |
| | December | 1 | 1 | 100.00 | 0 | 0.00 | 1 | 100.00 | 1 | 100.00 | 1 | 100.00 | 0 | 0.00 | 0 | 0.00 | 1 | 100.00 | 1 | 100.00 | 0 | 0.00 | 0 | 0.00 |

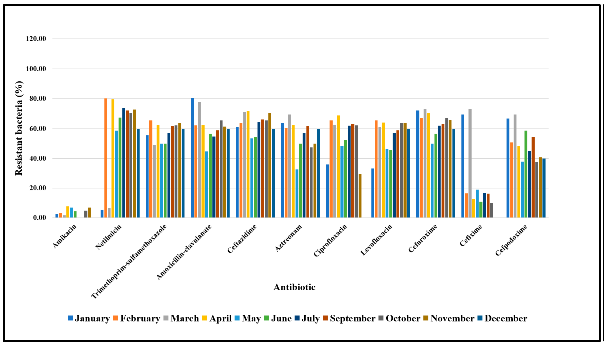


(A)

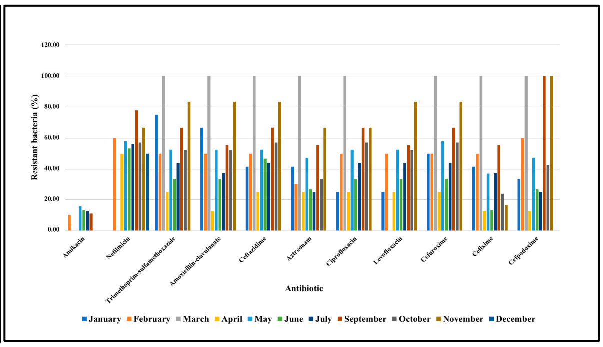


(B)

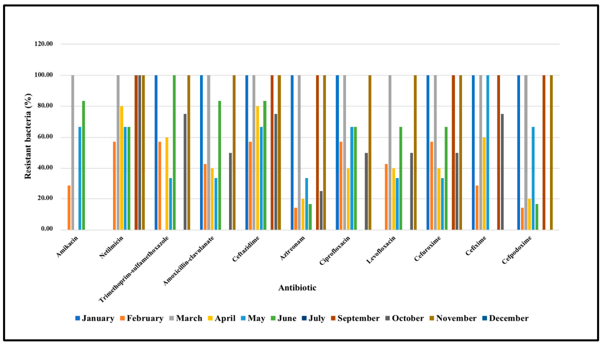


(C)

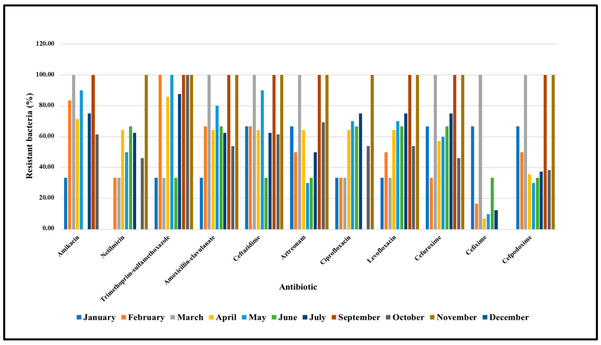


(D)

**Figure S1.** Graphical representation of trends in resistance rates of A) *E. coli* ;(B) *Klebsiella* spp.; (C) *Enterobacte*r spp.; (D) *Enterococcus* spp. over time (covering all months of 2018 except August).
